# Pleckstrin Levels Are Increased in Patients with Chronic Periodontitis and Regulated *via* the MAP Kinase-p38α Signaling Pathway in Gingival Fibroblasts

**DOI:** 10.3389/fimmu.2021.801096

**Published:** 2022-01-11

**Authors:** M. Abdul Alim, Duncan Njenda, Anna Lundmark, Marta Kaminska, Leif Jansson, Kaja Eriksson, Anna Kats, Gunnar Johannsen, Catalin Koro Arvidsson, Piotr M. Mydel, Tülay Yucel-Lindberg

**Affiliations:** ^1^ Department of Dental Medicine, Division of Pediatric Dentistry, Karolinska Institutet, Huddinge, Sweden; ^2^ Department of Dental Medicine, Division of Periodontology, Karolinska Institutet, Huddinge, Sweden; ^3^ Department of Microbiology, Faculty of Biochemistry, Biophysics and Biotechnology, Jagiellonian University, Kraków, Poland; ^4^ Department of Periodontology, Folktandvården Stockholms län AB, Folktandvården Eastmaninstitutet, Stockholm, Sweden; ^5^ Department of Clinical Science, Broegelmann Laboratory, University of Bergen, Bergen, Norway

**Keywords:** chronic periodontitis, pleckstrin, PLEK, inflammation, MAP kinase pathway, gingival fibroblasts, mPGES-1

## Abstract

Chronic periodontitis (CP) is a bacteria-driven inflammatory disease characterized by the breakdown of gingival tissue, the periodontal ligament, and alveolar bone, leading ultimately to tooth loss. We previously reported the pleckstrin gene (*PLEK*) to be highly upregulated in gingival tissue of patients with CP and the only gene concurrently upregulated in other inflammatory diseases including rheumatoid arthritis and cardiovascular diseases. Using saliva from 169 individuals diagnosed with CP and healthy controls, we investigated whether pleckstrin could serve as a novel biomarker of periodontitis. Additionally, we explored signal pathways involved in the regulation of *PLEK* using human gingival fibroblasts (HGFs). Pleckstrin levels were significantly higher (p < 0.001) in the saliva samples of patients with CP compared to controls and closely associated with CP severity. Immunohistochemical analysis revealed the expression of pleckstrin in inflammatory cells and gingival fibroblasts of CP patients. To explore the signal pathways involved in pleckstrin regulation, we stimulated HGFs with either interleukin-1β (IL-1β) or lipopolysaccharides (LPS) alone, or in combination with inhibitors targeting c-Jun N-terminal kinase, tyrosine kinase, protein kinase C, or p38 MAP kinase. Results showed that IL-1β and LPS significantly increased PLEK mRNA and pleckstrin protein levels. VX-745, the p38 MAP kinase inhibitor significantly decreased IL-1β- and LPS-induced pleckstrin levels at both the mRNA and the protein level. Together, these findings show that pleckstrin could serve as a salivary biomarker for the chronic inflammatory disease periodontitis and a regulator of inflammation *via* the p38 MAP kinase pathway.

## Introduction

Chronic periodontitis (CP) is an inflammatory disease driven by the host immune response to a microbiota shift (1, 2). The severe form of the disease affects up to 11%−15% of the human population and prevalence tends to increase with age (3, 4). Its hallmarks include progressive breakdown of the gingival tissue followed by destruction of periodontal ligament structures and alveolar bone (5, 6).

In the last decade, several studies have used microarray and RNA sequencing technology to describe the transcriptomic profile of CP in order to investigate molecules that could serve as reliable biomarkers for periodontitis (7–11). Frequently reported genes that have been shown to be significantly upregulated in CP include inflammation-related genes such as the interleukin family (e.g., *IL-1β*, *IL-8*, *IL-10 receptor*, *IL-12A*, *IL-17*, and *IL-19)* and the matrix metalloproteinases (e.g., *MMP-1*, *MMP-7*, and *MMP-9*); the membrane-spanning 4-domains family, subfamily A1 (*MS4A1*); complement 3 (*C3*); and the chemokine family (e.g., *CXCL-3* and *CXCL-12*) (7, 8, 10–12). Numerous studies have also reported a correlation between CP and other chronic inflammatory diseases, including cardiovascular disease (CVD), rheumatoid arthritis (RA), and diabetes (13, 14). To our knowledge, the networking link between periodontitis and other chronic inflammatory systemic diseases has not yet been identified. Our group, however, has previously reported the *PLEK* gene to be upregulated in CP compared with non-CP (fold change ~1.6) as well as commonly upregulated in individuals with ulcerative colitis (UC), CVD, and RA (12).

*PLEK* gene expression leads to the synthesis of pleckstrin. Pleckstrin is a 47-kDa protein mainly localized in the cytosol (15) but can be translocated to the cell membrane, in either its phosphorylated or unphosphorylated form (16). Pleckstrin was first discovered in platelets as a substrate for protein kinase C (PKC) enzymes, and it is involved in cytoskeletal reorganization, promoting cell-cell adhesion, and migration (17–19). PKC enzymes (15 isoenzymes in humans) (20) play a fundamental role in mitogen-activated protein kinase (MAPK) pathways that include phosphorylation of kinases involved in the c-Jun N-terminal kinase (JNK) and p38α (MAPK14) signal transduction pathways (21). In addition, PKC enzymes are also involved in the phosphorylation of kinases that lead to the downstream activation of nuclear factor kappa beta (NF-κβ) (22). To our knowledge, little has been published on the role of PKC in the regulation of pleckstrin levels in CP and on the levels, regulation, and signaling pathways of pleckstrin in other chronic inflammatory diseases. We previously reported that *PLEK* gene expression is upregulated in the gingival tissues of patients with periodontitis compared to healthy controls, as well as induced in response to LPS treatment in HGFs (12). In addition, using bioinformatics analyses, Song et al. identified the following genes involved in the regulation and progression of periodontitis: *PLEK*, cathepsin S (*CTSS*), interferon regulatory factor 4 (*IRF4*), and prostaglandin-endoperoxide synthase 2 (*PTGS2*) (23).

Based on our earlier findings, the present study on patients with CP assessed the biomarker potential of pleckstrin salivary levels for CP. Moreover, we explored the interlink or sub-cellular localization of pleckstrin with microsomal prostaglandin E synthase-1 (mPGES-1), an important key enzyme of inflammation processes involved in numerous chronic inflammatory diseases including RA, osteoarthritis, and periodontitis (24, 25). To our knowledge, this is the first study to demonstrate that pleckstrin levels in patients with CP are elevated compared with healthy subjects and that pleckstrin colocalizes with mPGES-1.

Finally, we performed *in vitro* functional studies to elucidate the signal transduction pathway(s) involved in pleckstrin regulation using human gingival fibroblasts, which are the most abundant cell type in gingival connective tissue.

## Materials and Methods

### Ethics Consideration

Ethical permits for this study were approved by the Regional Ethics Board in Stockholm (with reference numbers 2008/1935-31/3; 2014/1588-32/3; 2013/790‐31/2) and written informed consent was obtained from all individuals from whom patient material (including saliva samples and gingival biopsies) were collected.

### Collection of Saliva Samples and Detection of Pleckstrin Levels

Two cohorts of participants (n=169) were included in this study. For the first cohort, stimulated saliva samples were collected from 120 patients diagnosed with CP (n=63) and healthy controls (n=57). The mean age ± SD and (range) for the patients with periodontitis was 58.8 ± 12.7 (22–86), and for the healthy control group 42.3 ± 13.5 (24–77) years. The ratio of males/females were 31/32 in the periodontitis group and 22/35 in the healthy control group. The clinical diagnosis of CP was based on tooth sites with probing pocket depth ≥ 6 mm, bleeding on probing >30%, clinical attachment level ≥ 5 mm and radiographic evidence of bone loss. For the second cohort (n=49), stimulated saliva samples were collected from healthy controls and patients with different severity of periodontitis, based on radiographic evidence of marginal bone loss and sites bleeding on probing with pocket depth > 4mm, and healthy control group (n=10). For this cohort, the mean age ± SD (range) for patients with periodontitis was 69.5 ± 8.0 (51–83) and for the healthy group 64.4 ± 15.5 (31–81) years. The participants included in the control groups showed no marginal bone loss, probing depth ≤ 3.0 mm and bleeding on probing < 10%.

Briefly, all participants fasted 2 h prior to the clinical examination. Masticatory stimulation was induced by chewing paraffin tablets for 2 minutes before saliva was collected in pre-labelled 50 ml sterile falcon tubes. After collection, the saliva samples were immediately frozen at −20°C until further processing as previously described (26). The samples where then thawed and centrifuged at 500 x g for 10 min at 5°C and the supernatants were stored at −80°C until analysis.

Saliva samples were analyzed using the Human PLEK (pleckstrin) ELISA assay kit with a range of 0.156-10 ng/ml and sensitivity of 0.094 ng/ml (catalog # EH11196, Wuhan Fine Biotech Co, China). The ELISA assay was executed following the manufacturer’s protocol.

### Immunohistochemical and Immunofluorescence Staining of Gingival Tissues

To investigate the expression and localization of pleckstrin in human gingival tissue biopsies, we used immunohistochemistry and immunofluorescence techniques. Immunohistochemistry analysis on gingival tissue samples obtained from patients with CP was performed as previously described (12, 27). Briefly, sections (4 μm thick) were deparaffinized prior to treatment with antigen retrieval citrate buffer, pH 6. The Cell and Tissue Staining Kit (catalog # CTS005, R&D Systems, Minneapolis, USA) was used for the blocking steps, secondary antibody and chromogenic staining following the manufacturers protocol. Primary mouse anti-human pleckstrin antibody (1:50, Abcam, Cambridge, UK) was applied overnight. All washing steps and incubations with the primary antibody were performed in PBS with 0.1% saponin. Tissue sections were blocked with 5% normal goat serum after aspiration of the primary antibody and later incubated with biotinylated anti- mouse secondary antibody (R&D Systems, Minneapolis, USA). Tissue sections were developed by 3,3′-Diaminobenzidine (DAB) and counterstained with Mayer’s Hematoxylin (Sigma Aldrich, USA) before image visualization using a standard light microscope. For immunofluorescence, staining was performed following the protocol as described (28, 29) with some modification. Briefly, after primary antibody incubation (as described above), the tissue sections were washed in PBS (2 × 5 min) and then incubated for 60 min (on a shaker) with a mixture of biotinylated secondary antibodies including anti-rabbit conjugated with Alexa Fluor 594 and anti-mouse conjugated with Alexa Fluor 488 (1:1000 dilution in PBS-0.1% saponin). For visualization of nuclei, DAPI (4,6-diamidino-2-phenylindole, Invitrogen) staining was performed. The localization of pleckstrin in the tissue and cells was monitored by using confocal microscopy (Nikon Instruments, Melville, NY).) and the images were adjusted with the image J software (Fiji, ImageJ2). All photographs were taken at original magnifications of ×200 or ×400 objective.

### Cell Culture Experiments

HGFs were established from gingival tissue biopsies obtained from three healthy donors with no clinical signs of periodontitis, as previously described (30). The cells were seeded in 60 mm Petri dishes and maintained in Dulbecco’s Modified Eagle Medium (DMEM) supplemented with penicillin (50 units/ml), streptomycin (50 μg/ml), and 5% fetal calf serum (Life Technologies Europe BV) and cultured at 37°C for 24 h. Afterwards, the cells were cultured, at a final concentration of 0.3 x 10^6^ cells/2 ml, in DMEM containing 0.5 ng/ml IL-1β (R&D Systems, Minneapolis, USA) and/or 6.0 μg/ml Lipopolysaccharide (LPS) from *Porphyromonas gingivalis* (*In vivo* Gen, Toulouse, France) in the absence or presence of different signal pathway inhibitors by following the final concentrations of 1.0 μM phorbol-12-myristate-13-acetate (PMA) – PKC activator; 2.0 μM Bisindolylmaleimide I, Hydrochloride (BIS) – PKC inhibitor; 2.0 μM PD 153035 hydrochloride (PD) – tyrosine kinase epidermal growth factor receptor (EGFR) inhibitor; 20 μM SP600125 (SP) – c-Jun N-Terminal kinase inhibitor and 1.0 μM Neflamapimod (VX-745) – a highly selective inhibitor of p38α. All the inhibitors were purchased from Sigma-Aldrich (St. Louis, MO, USA). After 24 h incubation with or without inhibitors, the cells were used for flow cytometry analysis or the real-time polymerase chain reaction (qPCR) experiments. In addition, the cell culture supernatants were assessed for extracellular pleckstrin levels using the Human PLEK (pleckstrin) ELISA assay kit (catalog # EH11196, Wuhan Fine Biotech, China).

Peripheral blood mononuclear cells (PBMCs) were purified from buffy coats from healthy blood donors who had given their informed consent (Karolinska University Hospital Laboratory, Huddinge, Sweden) using density gradient centrifugation on Ficoll Paque Plus (GE Healthcare, Uppsala, Sweden). The CD14-positive fraction of PBMCs was isolated with CD14+ magnetic microbeads, according to the manufacturer’s instructions (Miltenyi Biotec, Bergisch Gladbach, Germany). PBMCs were cultured in 96‐well plates (NUNC, delta surface) in α‐MEM, supplemented with 10% FBS, GlutaMAX (2 mmol/L), streptomycin (100 μg/mL), penicillin (100 U/mL) (all from GIBCO, Grand Island, NY, USA). The cells were thereafter cultured, at a final concentration of 0.15 x 10^6^ cells/0.2 ml, in α‐MEM alone or in the presence of LPS for 6 days and the culture supernatants were harvested, centrifuged at 200 × g for 10 minutes, and stored at -80°C, for analyses of pleckstrin.

### Double Immunofluorescence Staining of Gingival Fibroblasts

To evaluate the sub-cellular localization of pleckstrin and mPGES-1, we further used immunofluorescence technique following confocal analysis. For immunofluorescence double staining, culture fibroblast cells were collected after trypsinization. The staining with antibodies was performed with some modification as described before (28, 29, 31). Briefly, the cells were first placed onto glass slides, allowed to settle for 15 minutes and the medium sucked out with Whitman filter paper. After that, cells were immediately fixed with cold acetone (-20°C) for 15 min. Next, cells slides were first blocked with 5% horse and/or goat serum, followed by PBS wash (2×). After the first blocking step, the cells were additionally blocked to quench nonspecific binding of avidin (see the final step) using cell and tissue staining kit (catalog # CTS005, R&D Systems, Minneapolis, USA). Cells were then incubated with a mixture of two primary antibodies overnight (4°C in the dark). The primary antibodies mixture was as mouse anti-human pleckstrin monoclonal antibody (1:50, Abcam, Cambridge, UK) and polyclonal rabbit anti-mPGES-1 (1:100; Cayman Chemical, Ann Arbor, MI). Both primary antibodies were diluted in PBS-0.1% saponin mix to permeabilize the cells. Next, the cells were washed in PBS (2 × 5 min) and then incubated for 60 min (on a shaker) with a mixture of biotinylated secondary antibodies including anti-rabbit conjugated with Alexa Fluor 594 and anti-mouse conjugated with Alexa Fluor 488 (1:1000 dilution in PBS-0.1% saponin). For visualization of nuclei, DAPI (4,6-diamidino-2-phenylindole, Invitrogen) staining was performed. The localization of pleckstrin in the cells was monitored by using confocal microscopy (Nikon Instruments, Melville, NY) and the images were adjusted with the image J software (Fiji, ImageJ2). All photographs were taken at original magnifications of ×400, or ×600 with an oil objective.

### RNA Isolation and RT-qPCR

Total RNA was isolated from treated and untreated HGFs cultures. Briefly, total RNA was extracted using the RNeasy Mini Kit (Qiagen, Valencia, CA, USA) according to the manufacturer’s instructions, and quantified using a Qubit spectrophotometer (Molecular Probes, Eugene, Oregon, USA). cDNA synthesis was performed from 0.5-1 μg of total RNA in a 20 μl reaction using the iScript™ cDNA Synthesis Kit (BioRad, Hercules, CA, USA), according to the manufacturer’s instructions. Gene expression analysis was performed by quantitative real-time PCR (qPCR) using *PLEK* TaqMan Gene Expression Assay (Hs00160164_m1, ThermoFisher Scientific, USA) together with TaqMan Universal PCR Master Mix (ThermoFisher Scientific, USA). All qPCR reactions were run in triplicates on the 7500 Fast Real-Time PCR system (Applied Biosystems, ThermoFisher Scientific, USA) using the following reaction conditions: 50°C for 2 minutes followed by 95°C for 10 minutes and then 40 cycles of 95°C for 15 seconds and annealing and extension at 60°C for 1 minute as previously described (12). The relative gene expression was calculated according to the ΔΔCt method, where the mean Ct for *PLEK* expression was normalized against the mean Ct of the housekeeping gene glyceraldehyde 3-phosphate dehydrogenase (*GAPDH*) expression.

### Flow Cytometry Experiments

Cells were collected by trypsinization (0.025% trypsin), washed with PBS and fixed in 2% paraformaldehyde before permeabilization with 0.1% saponin and 0.01M HEPES as previously described (32). Pleckstrin was detected using secondary Fluorescein isothiocyanate (FITC) labeled antibody bound to the pleckstrin primary antibody and analyzed on the FITC channel in the flow cytometer (BD FACSVerse, BD Biosciences, USA). For each sample a minimum of 10,000 events was acquired, and the gating strategy was determined using a viability control and side scatter area (SSA) versus forward scatter area (FSA) parameters. Single cells expressing pleckstrin were gated using the forward side scatter height versus the (forward scatter area). Analysis was performed using Flow Jo™ Software (Becton, Dickinson and Company, USA) and graphical representation was done using GraphPad Prism v8.4.2 (GraphPad Software, San Diego CA, USA).

### Statistical Analysis

Descriptive statistics were performed for pleckstrin levels in saliva samples collected from patients with periodontitis and control subjects without periodontitis. For differences between pleckstrin levels in the saliva samples of patients with periodontitis compared with controls, statistical significance was calculated using Mann Whitney test and p-value ≤ 0.05 was considered statistically significant (*p ≤ 0.05; **p ≤ 0.01 and ***p ≤ 0.001). The statistical analysis and graphical representation were carried out using the Statistical Package for the Social Sciences (SPSS Statistics 26.0; SPSS Inc) and GraphPad Prism v8.4.2 (GraphPad Software, San Diego CA, USA). Multiple linear regression analysis was used with the concentrations of pleckstrin as dependent variable. In order to achieve normality, the pleckstrin concentrations were first log-transformed. Periodontitis, age and sex were included as independent variables. Pearson’s correlation coefficients were used to determine associations between the log-transformed concentrations of pleckstrin and periodontal variables. For cell culture experiments, results were presented as mean values ± SD from at least three independent experiments and one-way ANOVA with Tukey’s multiple comparisons test was used to determine the statistical significance for comparable treatments. *p ≤ 0.05; **p ≤ 0.01 and ***p ≤ 0.001.

## Results

### Pleckstrin Levels in Saliva Samples of Patients With Chronic Periodontitis

**Figure 1:**
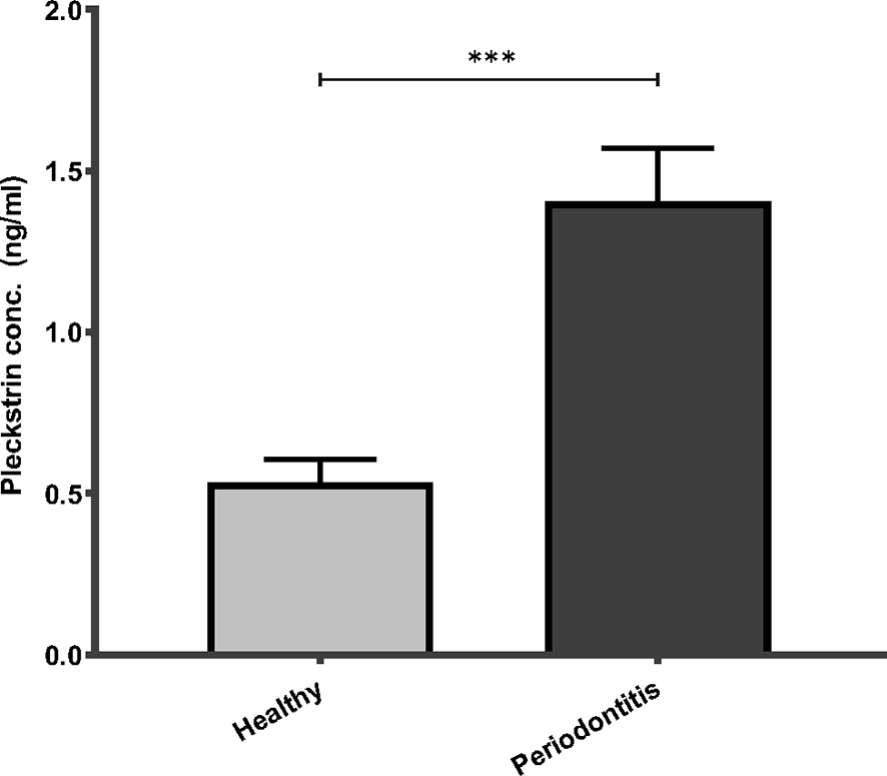
Pleckstrin levels in saliva samples of patients with CP and controls. Bar plot showing salivary pleckstrin concentrations (ng/ml) of patients with CP compared with healthy controls. Data are presented as the mean values ± SEM. Significance was calculated following Mann Whitney test. ***p-value < 0.001. CP, Chronic periodontitis.

Pleckstrin levels were measured in the stimulated saliva samples of two cohorts (first cohort: n=120, second cohort: n=49). Pleckstrin levels in saliva samples of patients with CP were significantly higher (p < 0.001) compared to the control group as shown in **Figure 1**. The mean level of pleckstrin (ng/ml ± SEM) in the CP group was 1.40 ± 0.17 and in the control group, 0.54 ± 0.07 (**Figure 1**). The age difference between the two groups was significant (p < 0.01) with a higher mean age in the CP group (58.8 ± 12.7 years) versus the healthy group (42.2 ± 13.5). However, multiple linear regression analysis showed that pleckstrin concentrations differed significantly (p=0.003) between the two groups irrespective of age and gender (**Table 1**).

**Table 1:** Multiple linear regression analysis using the log-transformed concentrations of pleckstrin as the dependent variable. R^2 =^ 0.38 (n=120).

Variable	B	S.E.	p
Periodontitis (0=no, 1=yes)	0.526	0.175	0.003
Age (years)	0.020	0.006	<0.001
Gender (0=Male, 1=Female)	-0.632	0.152	<0.001

B, Unstandardized beta; S.E., Standard error of unstandardized beta.

In the second cohort, pleckstrin levels were significantly and positively correlated with bleeding on probing and with the number of sites bleeding on probing with pocket probing depth > 4 mm or > 6 mm (**Table 2**).

**Table 2:** The correlations (Pearson correlation coefficients) between the log-transformed pleckstrin concentrations (ng/ml) and the periodontal variables bleeding on probing index and number of sites bleeding on probing with pocket depth > 4 mm or > 6 mm (n=49).

Variable	Pearson correlation	p
Bleeding on probing index	0.35	0.014
Number of sites bleeding on probing with pocket depth > 4 mm	0.40	0.004
Number of sites bleeding on probing with pocket depth > 6 mm	0.38	0.006

### Pleckstrin Expression in Gingival Tissue of Patients With Chronic Periodontitis

**Figure 2:**
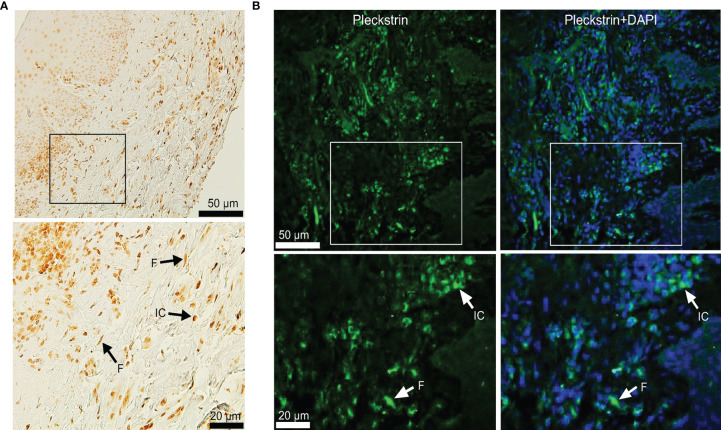
Immunohistochemical staining of Pleckstrin expression in gingival tissue biopsies. **(A)** Representative histological staining of gingival tissue of patients with CP stained with DAB showing the detection of pleckstrin in gingival inflammatory cells and fibroblasts (black arrows in the highlighted area). **(B)** Immunofluorescence staining of pleckstrin in human gingival biopsy. The confocal images show the localization of pleckstrin (green) with a DAPI (blue) staining. DAPI was used as a nuclear staining of tissue cells. The square areas were highlighted below the images with inflammatory cells (IC) and fibroblasts (F) for pleckstrin positive staining (white arrows). Scale bars: 20 μm and 50 μm. CP, Chronic periodontitis.

To investigate pleckstrin protein expression in the gingival tissue of patients with CP, we sectioned the gingival tissues and evaluated tissue characteristics using immunostaining followed by immunofluorescence detection with a confocal imaging (**Figure 2**). Immunohistochemical and immunofluorescence staining showed the extent of pleckstrin occurrence in gingival tissue samples of patients with CP. Microscopic images of gingival tissue sections showed immune cells, endothelial cells, and fibroblasts positively stained for pleckstrin (**Figures 2A, B** **)**.

### *PLEK* mRNA Levels in Human Gingival Fibroblasts Stimulated With IL-1β or LPS

**Figure 3:**
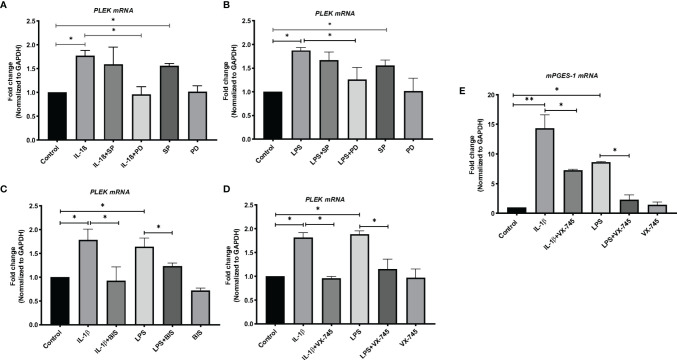
Bar graphs of relative mRNA levels (fold change) of *PLEK* and mPGES-1 by qPCR. **(A)** *PLEK* mRNA levels in HGFs (0.3x10^6^ cells/ml) stimulated with IL-1β (500 pg/ml) for 24 h alone or in combination with SP (20 μM) or PD (2 μM). **(B)** *PLEK* mRNA levels in gingival fibroblasts stimulated with *P. gingivalis* LPS (5μg/ml) alone or in combination with SP or PD. **(C)** *PLEK* mRNA levels in HGFs stimulated with IL-1β or LPS alone or in combination with BIS. **(D)** Gingival fibroblast cells were stimulated with IL-1β and/or LPS alone or in combination with VX-745 (1.0 µM) for 24 h, followed by measurement of relative mRNA levels (fold change) of *PLEK*. **(E)** *mPGES-1* mRNA level after IL-1β and LPS treatment alone or in combination with VX-745. The relative mRNA levels were normalized to the levels of the housekeeping gene GAPDH. Data are presented as mean values ± SD from at least three independent experiments. Significance was calculated with one-way ANOVA with Tukey’s multiple comparisons test where p-values were set as *p ≤ 0.05 and **p ≤ 0.01. SP, SP600125 (inhibitor of c-Jun N-terminal kinase JNK); PD, PD 153035 hydrochloride (inhibitor of the epidermal growth factor receptor tyrosine kinase EGFR); BIS, Bisindolylmaleimide I, Hydrochloride (Protein kinase C inhibitor); VX-745 , Neflamapimod (selective inhibitor p38α MAPK inhibitor).

The next set of experiments studied the expression of *PLEK* at the mRNA level in human primary gingival fibroblasts using qPCR. Stimulation of HGFs with the inflammatory mediators IL-1β or LPS (of *P. gingivalis*) significantly increased *PLEK* mRNA levels compared to control cells treated with medium alone (**Figures 3A–D**).

We used mPGES-1 as a positive control since this inflammatory enzyme is expressed in gingival tissue of patients with periodontitis as well as induced by inflammatory mediators and abolished by a variety of signal pathways inhibitors in HGFs (30). Similar to *PLEK* mRNA expression, both IL-1β and LPS treatment also increased the mPGES-1 mRNA expression in the cells (**Figure 3E**).

### Effects of JNK and Tyrosine Kinase Inhibitors on IL-1β- and LPS-Induced *PLEK* mRNA Levels

To investigate the signal transduction pathways involved in the regulation of *PLEK* expression, we performed additional *in vitro* experiments using the JNK inhibitor (SP) and the tyrosine kinase EGFR inhibitor (PD). The cells were treated with the proinflammatory mediators IL-1β or LPS, either alone or in combination with signal pathway inhibitors. The qPCR analysis revealed that SP treatment did not affect IL-1β or LPS-induced *PLEK* mRNA levels in HGFs (**Figures 3A, B** **)**. SP alone, however, significantly increased *PLEK* transcripts compared with unstimulated control cells. On the other hand, PD, in combination with IL-1β or LPS, significantly decreased both IL-1β- and LPS-induced *PLEK* mRNA levels compared to treatment with PD alone (**Figures 3A, B** **)**. These results indicate that the tyrosine kinase signaling pathway might regulate inflammation-induced *PLEK* expression and that the JNK kinase pathway might regulate the constitutive *PLEK* expression.

### Effects of BIS, the PKC Inhibitor, on IL-1β- or LPS-Induced *PLEK* mRNA Levels

To investigate the involvement of PKC in the regulation of *PLEK*, we cultured gingival fibroblasts with BIS (the PKC inhibitor) in the absence or presence of IL-1β or LPS. Treatment of HGFs with BIS in combination with IL-1β or LPS reduced IL-1β- and LPS-induced *PLEK* mRNA levels compared to IL-1β or LPS treatment alone (**Figure 3C**). Treatment with BIS alone did not significantly change *PLEK* mRNA levels compared to unstimulated control cells.

Furthermore, we observed that PMA, known to activate PKC, slightly induced *PLEK* mRNA levels in HGFs, while pleckstrin protein levels were unaffected (**Supplementary Table 1**).

### Effects of p38α Inhibitor on IL-1β- and LPS-Induced *PLEK* mRNA Levels

To further explore the regulation of *PLEK*, we also investigated the effects of a selective p38α MAP kinase inhibitor (VX-745) on inflammation-induced *PLEK* mRNA levels. Simultaneous treatment of the cells with VX-745, in combination with IL-1β or LPS, significantly inhibited both IL-1β-induced as well as LPS-induced mRNA levels of *PLEK* (**Figure 3D**). VX-745 alone did not affect *PLEK* mRNA levels (**Figure 3D**). Similar to *PLEK*, p38α MAP kinase inhibitor significantly reduced IL-1β- and LPS-induced mRNA levels of *mPGES-1* in HGFs (**Figure 3E**). These findings suggest that the MAPK-p38α signaling pathway may be involved in the regulation of *PLEK* expression.

### Effects of Signal Pathway Inhibitors on Intracellular Pleckstrin Levels in HGFs

**Figure 4:**
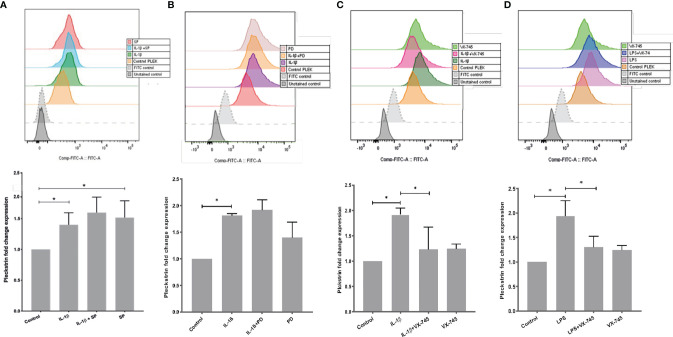
Intracellular pleckstrin detection in HGFs analyzed by Flow cytometry. **(A)** Overlay histogram profile and corresponding bar graph plot (shown below) showing intracellular pleckstrin fold change expression in response to IL-1β treatment alone or in combination with SP in gingival fibroblasts. **(B)** Overlay histogram profile and corresponding bar graph plot (below) showing intracellular pleckstrin fold change expression after IL-1β treatment alone or in combination with PD in gingival fibroblasts. **(C)** Overlay histogram profile and corresponding bar graph plot (below) showing intracellular pleckstrin fold change expression after IL-1β treatment alone or in combination with VX-745 in gingival fibroblasts. **(D)** Overlay histogram profile and corresponding bar graph plot (below) showing intracellular pleckstrin fold change expression after LPS treatment in the absence or presence of VX-745 in gingival fibroblasts. Data are presented as the mean values ± SD from at least three independent experiments. Significance was calculated with one-way ANOVA with Tukey’s multiple comparisons test where p-values were set as *p ≤ 0.05. SP SP600125 (inhibitor of c-Jun N-terminal kinase JNK); PD, PD 153035 hydrochloride (inhibitor of the epidermal growth factor receptor tyrosine kinase EGFR); VX-745, Neflamapimod (selective inhibitor p38α MAPK inhibitor). Raw data images and gating strategies can be found in **Supplementary Figure 3**.

Using flow cytometry, we explored intracellular protein levels of pleckstrin in HGFs. After 24h stimulation with IL-1β, intracellular pleckstrin levels were significantly increased (**Figures 4A–C**). Treatment of the cells with IL-1β, in combination with either SP or PD, did not significantly affect IL-1β-induced (**Figures 4A, B** **)** or LPS-induced levels of pleckstrin (Figure not shown). However, similar to mRNA upregulation, SP alone significantly (p < 0.05) upregulated intracellular protein levels of pleckstrin compared to control cells (**Figure 4A**).

Additionally, we quantified (fold change) the effect of VX-745 on IL-1β- and LPS-induced pleckstrin. VX-745 significantly inhibited upregulation of pleckstrin by IL-1β and LPS. Alone, however, VX-745 had no effect on protein levels (**Figures 4C, D** **)**. These results suggest that the MEK3-p38α signaling pathway may regulate pleckstrin since VX-745, a potential and highly selective inhibitor of p38α MAP kinase, inhibited pleckstrin expression at both the mRNA and protein levels. Moreover, in agreement with pleckstrin protein levels, VX-745 reduced the IL-1β-induced mPGES-1 expression in HGFs (**Supplementary Figure 1**).

### Effects of Signal Pathway Inhibitors on IL-1β- or LPS-Induced Pleckstrin Production in HGF

**Figure 5:**
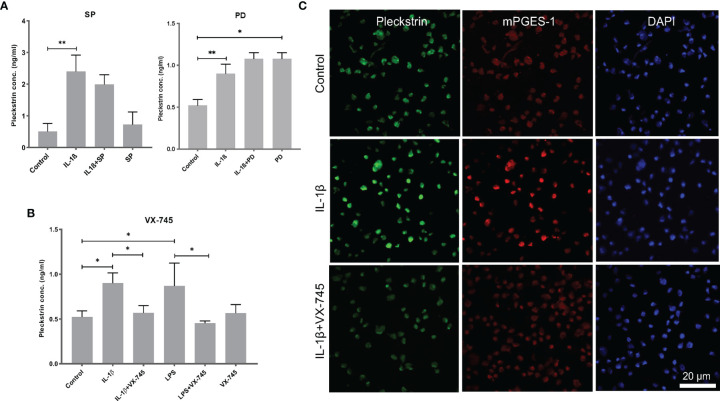
Extracellular pleckstrin detection in HGFs using ELISA. **(A)** Bar plot demonstrating extracellular pleckstrin levels in HGFs stimulated with IL-1β and/or LPS treatment alone or in combination with signal transduction pathway inhibitors SP and PD. **(B)** Bar plot showing extracellular pleckstrin production after IL-1β or LPS treatment alone or in combination with p38α MAPK inhibitor VX-745 in gingival fibroblasts. **(C)** Immunofluorescence staining of pleckstrin and mPGES-1 expression after IL-1β treatment for 24 h alone or in combination with p38α MAPK inhibitor VX-745 in gingival fibroblast cells. The confocal images depict the localization of pleckstrin (green) and mPGES-1 (red) with DAPI (blue) staining which was used as a nuclear marker. Isotype or unstained controls for pleckstrin and mPGES-1 staining are used for revealing the specificity of the staining (data not shown here). Scale bars: 20 μm. Data are presented as the mean values ± SD from at least three independent experiments. Significance was calculated with one-way ANOVA with Tukey’s multiple comparisons test where p-values were set as *p ≤ 0.05 and **p ≤ 0.01. SP, SP600125 (inhibitor of c-Jun N-terminal kinase JNK); PD, PD 153035 hydrochloride (inhibitor of the epidermal growth factor receptor tyrosine kinase EGFR); VX-745, Neflamapimod (selective inhibitor p38α MAPK inhibitor).

We used ELISA to measure pleckstrin levels in the supernatants of the HGF cultures after 24h incubation with IL-1β or LPS in the presence of various inhibitors. Pleckstrin production increased significantly (p < 0.01) in response to treatment with either IL-1β or LPS (**Figures 5A, B** **)**. The signal pathway inhibitors of JNK (SP) and tyrosine kinase (PD), alone or in combination with IL-1β, had no effect on pleckstrin secretion. Similarly, neither SP or PD affected LPS upregulation of pleckstrin levels significantly when compared to treatment with LPS alone or to untreated control cells (Figure not shown).

Interestingly, the pleckstrin protein levels upregulated by IL-1β or LPS were significantly reduced in the presence of VX-745 (an MEK3-p38α inhibitor). VX-745 alone did not affect pleckstrin levels compared to control cells (**Figure 5B**). Confocal image analysis demonstrated that VX-745 reduced pleckstrin levels during costimulation with IL-1β (**Figure 5C**).

### Pleckstrin Levels in Human Peripheral Blood Mononuclear Cells

Due to the presence of pleckstrin in inflammatory cells (**Figures 2A, B** **)**, we decided to culture peripheral blood mononuclear cells (PBMCs) with *P. gingivalis* LPS. Pleckstrin levels were significantly (p < 0.05) increased in response to LPS treatment compared to untreated control PBMCs (**Supplementary Figure 2**).

### Colocalization of Pleckstrin and mPGES-1 After IL-1β or LPS Induction

**Figure 6:**
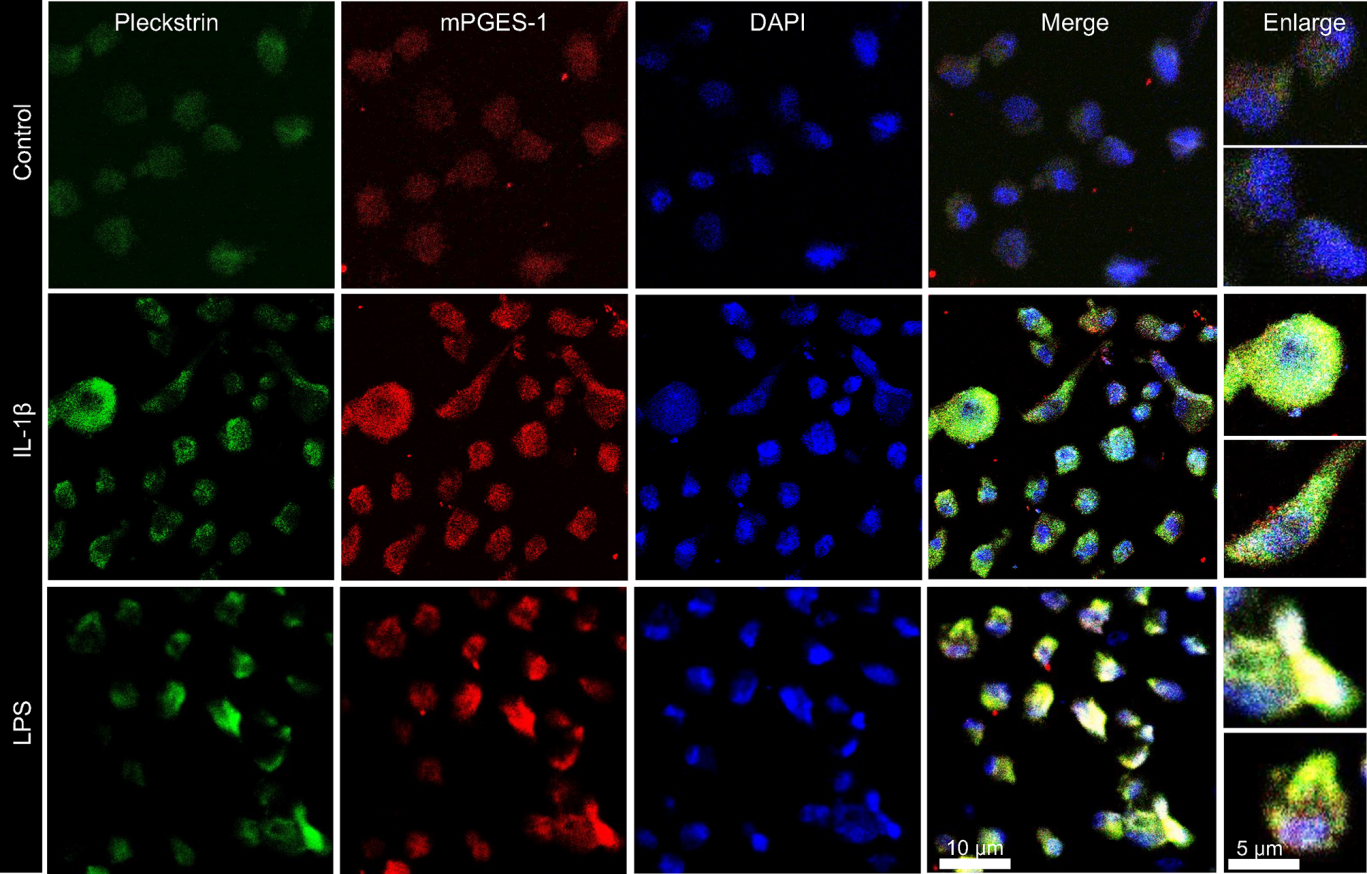
Visualization and co-localization of pleckstrin protein with mPGES-1 in unstimulated control and IL-1β or LPS stimulated HGFs using confocal microscopy. Immunofluorescence staining of fibroblast cells showing co-localization of pleckstrin (green) with mPGES-1 (red), an inflammatory marker, and DAPI (blue), a nuclear marker. Scale bars: 10 μm and 5μm.

In the final set of experiments, we explored the coexpression of pleckstrin with mPGES-1, an inflammation-induced enzyme previously reported to be expressed in periodontal tissue. **Figure 5C** shows how cellular IL-1β-induced pleckstrin and IL-1β-induced mPGES-1 levels are reduced in the presence of VX-745. We also observed partial colocalization of pleckstrin with mPGES-1 (**Figure 6**).

## Discussion

Pleckstrin has been implicated in various autoimmune and inflammatory diseases. Our previous transcriptomic analysis showed that *PLEK* is significantly upregulated in the gingival tissue samples of patients with CP compared to healthy controls (12). These analyses also identified *PLEK* as the only gene commonly expressed in the chronic inflammatory diseases CVD, RA, UC, and CP (12). In the present study, we report that protein levels of pleckstrin are significantly higher in saliva samples from patients with CP compared to healthy controls. In addition, we also report that pleckstrin levels are significantly associated with periodontal variables, including bleeding on probing and number of bleeding on probing sites with probing pocket depth > 4 mm or > 6 mm, reflecting the severity of the disease.

Pleckstrin, also known as P47-phosphoprotein, is involved as a substrate for PKC in platelets and leukocytes (33). This protein, involved in various adaptive immune responses, has been detected in peripheral blood lymphocytes, monocytes, granulocytes, and cultured leukemic cells (34, 35). Notably, associations between pleckstrin and inflammatory diseases like UC, celiac disease, diabetes, RA and periodontitis have been reported (12, 23, 36–38), although the underlying pathophysiological mechanisms are unclear. The Ding et al. study on diabetes demonstrated that phosphorylation of pleckstrin (a 47-kDa protein) is significantly increased in mononuclear phagocytes from diabetics compared with controls (36). In that study, the authors also used RNA interference silencing to demonstrate that pleckstrin phosphorylation is an essential intermediate step in the production and activation of the signaling pathways of proinflammatory cytokines (36). The Medrano et al. study on patients with UC and celiac disease, autoimmune diseases, found *PLEK* expression to be upregulated in tissue biopsies from the colon and rectum (37). Moreover, He et al. found pleckstrin homology (PH) domain-containing family O member 1 to be highly expressed in osteoblasts, which suggests that it contributes to joint inflammation in patients with RA (39). The immunohistochemical findings in the present study showed higher pleckstrin levels in gingival tissue biopsies from patients with CP when compared to healthy controls, in agreement with Lundmark et al. (12) Using microarray data on healthy and specimens of periodontitis-affected gingival tissue (downloaded from the Gene Expression Omnibus database), Song et al. identified an involvement of *PLEK* in the development and progression of periodontitis, which is consistent with our findings (23). Thus, our novel findings of significant differences in salivary levels of pleckstrin between patients with CP and healthy controls implies that this protein could be a non-invasive diagnostic biomarker for periodontitis.

We also performed *in vitro* functional studies to investigate the effects of IL-1β and LPS (of *P. gingivalis*) on mRNA and pleckstrin protein levels in HGFs, which are the predominant cells in gingival connective tissue. The significant upregulation of pleckstrin levels in the presence of IL-1β or LPS in gingival cells further supports a potential role of this protein in the inflammatory response driving periodontal disease progression. Both IL-1β, found to be elevated in salivary and gingival fluid samples of patients with CP (40, 41), and LPS of *P. gingivalis*, found frequently in the periodontal pockets of patients with CP (42), can stimulate production of pleckstrin in gingival cells. Since pleckstrin protein levels were also increased in LPS-stimulated PBMCs, additional findings of our *in vitro* studies, suggests that these cells collectively may also contribute to increased expression of Pleckstrin and thereby contribute to the increased levels of Pleckstrin detected in saliva samples.

To explore the signal pathways involved in the regulation of pleckstrin, we decided, using an inhibitor (BIS) or an activator (PMA) of PKC, to first determine the involvement of this enzyme. As expected, BIS lowered inflammation-induced *PLEK* mRNA levels in HGFs, whereas the phorbol ester of PMA raised mRNA levels. At the protein level, however, neither BIS nor PMA affected pleckstrin concentrations. According to Ding et al. rapid phosphorylation of pleckstrin (the PKC substrate) in response to a different agonist, such as phorbol ester, may also occur downstream of PKC activation, which might explain the lack of BIS or PMA effect on pleckstrin concentrations (36). The PH domain is a protein that has been identified in several proteins involved in signal transduction pathways; Harlan et al. have suggested that these domains bind to phosphatidylinositol- 4,5-bisphosphate (43). Furthermore, PH domains may be involved in protein-protein interactions including numerous PKC isoforms, which have been reported to interact with the PH domains of Akt, a serine/threonine-specific protein kinase, and the cytoplasmic form of tyrosine kinases (44, 45).

Next, we evaluated the involvement of the JNK and tyrosine kinase pathways in inflammation-induced *PLEK* expression by stimulating gingival cells with IL-1β or LPS in combination with various pathway inhibitors to target the JNK and tyrosine kinase pathways. As neither SP nor PD affected inflammation-induced pleckstrin levels, in contrast to control cells, it might be that intracellular regulation of pleckstrin in HGFs does not involve the JNK and tyrosine kinase signaling pathways in IL-1β- or LPS-stimulated cells. An explanation for this observation may be that the MAPK signaling pathway comprises several sub-pathways, including ERK 1/2 and p38 MAPK, which may also regulate pleckstrin levels in response to cytokine-induced inflammation (46, 47). Thus, one or more of these sub-pathways may have normalized any action of the JNK and tyrosine kinase pathways. The difference in results for SP and PD between the FACS flowmeter and ELISA might indicate that the JNK pathway regulates intracellular pleckstrin levels while the tyrosine kinase pathway regulates pleckstrin secretion levels.

To further explore the role MAPK signaling pathway, we also studied the effects of VX-745, a selective p38α MAP kinase inhibitor, on inflammation-induced *PLEK* mRNA levels and pleckstrin protein levels. Our finding that treatment of HGFs with VX-745 in combination with IL-1β or LPS significantly inhibited levels of both mRNA and pleckstrin protein suggests that the p38α MAPK signaling pathway may be involved. Plotnikov et al. have documented the central role that the MAPK pathway plays in signal transduction whereby extracellular stimuli are converted into a wide range of cellular responses including inflammatory responses, stress responses, proliferation, differentiation, and apoptosis survival (48). Studies have extensively explored the signaling pathways of p38 MAPK as a molecular target for the inhibition of chronic inflammation (49, 50), and Kirkwood et al. have described the potential beneficial effects of p38 inhibitors on LPS-induced alveolar bone loss and periodontitis (51).

## Conclusions

In the present proof-of-concept study, we demonstrated that pleckstrin levels are higher in saliva samples of patients with CP than in healthy individuals, suggesting that pleckstrin has the potential to be a novel salivary biomarker of CP that can be easily assessed using non-invasive sampling techniques. A larger cohort than the one used in this study would be required to validate its potential as a biomarker for the diagnosis and monitoring of various stages of periodontitis and its associated diseases, including cardiovascular diseases and rheumatoid arthritis. Although our results also indicate that pleckstrin is regulated *via* the MEK3- p38α pathway, further studies are required to elucidate the exact signaling pathways involved in pleckstrin regulation in order to devise future treatment strategies for periodontitis and periodontitis-associated diseases.

## Data Availability Statement

The original contributions presented in the study are included in the article/supplementary material. Further inquiries can be directed to the corresponding author.

## Ethics Statement

The studies involving human participants were reviewed and approved by the regional ethics board in Stockholm (reference numbers 2008/1935-31/3; 2014/1588-32/3; 2013/790‐31/2). The patients/participants provided their written informed consent to participate in this study.

## Author Contributions

TY-L and AL conceived the project and designed the study. LJ, KE, GJ, and AK contributed to recruitment of study participants, performed clinical analyses, and collected the saliva samples. MAA, DN, AL, MK, and CA performed the cell experiments and immunohistochemistry analyses and MAA performed immunofluorescence staining. LJ, MAA, and DN carried out the statistical analyses. MAA, DN, TY-L, and PM prepared the original draft. All authors contributed to the article and approved the submitted version.

## Funding

This study was supported by grants from the Swedish Research Council (2017-02084); the Patent Revenue Fund for Research in Preventive Odontology; the steering group KI/Region Stockholm for dental research (SOF); the Broegelmann Foundation; the Norwegian Research Council (296129); and Karolinska Institutet, Stockholm, Sweden.

## Conflict of Interest

The authors declare that the research was conducted in the absence of any commercial or financial relationships that could be construed as a potential conflict of interest.

## Publisher’s Note

All claims expressed in this article are solely those of the authors and do not necessarily represent those of their affiliated organizations, or those of the publisher, the editors and the reviewers. Any product that may be evaluated in this article, or claim that may be made by its manufacturer, is not guaranteed or endorsed by the publisher.
