# Supplementary figures and images for: Pleckstrin Levels Are Increased in Patients with Chronic Periodontitis and Regulated via the MAP Kinase-p38α Signaling Pathway in Gingival Fibroblasts
Source: Front Immunol. 2022 Jan 11;12:801096. doi: 10.3389/fimmu.2021.801096 (PMC8787058; doi:10.3389/fimmu.2021.801096)

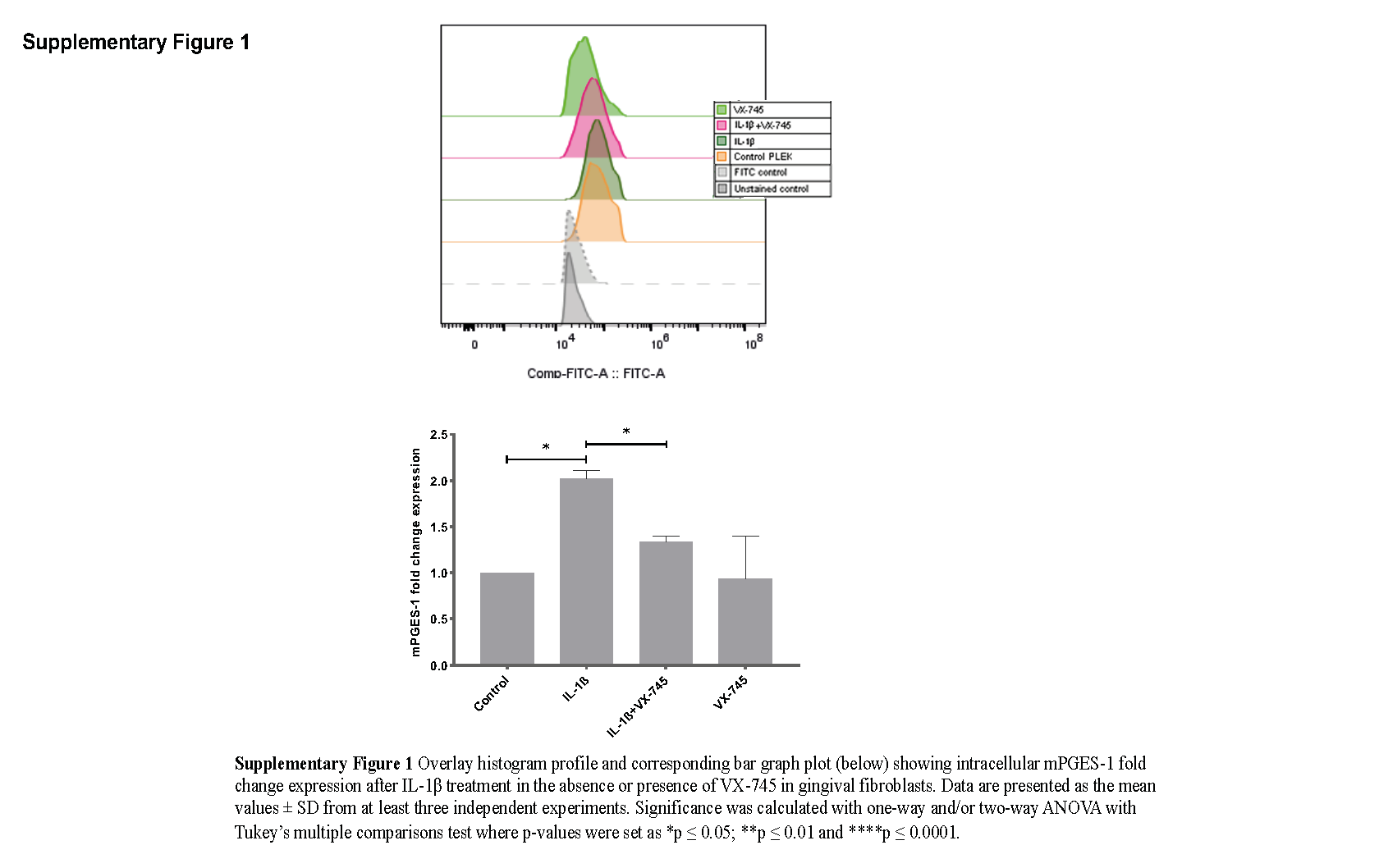

Supplement: Supplementary file 1 [file Image_1.tiff]

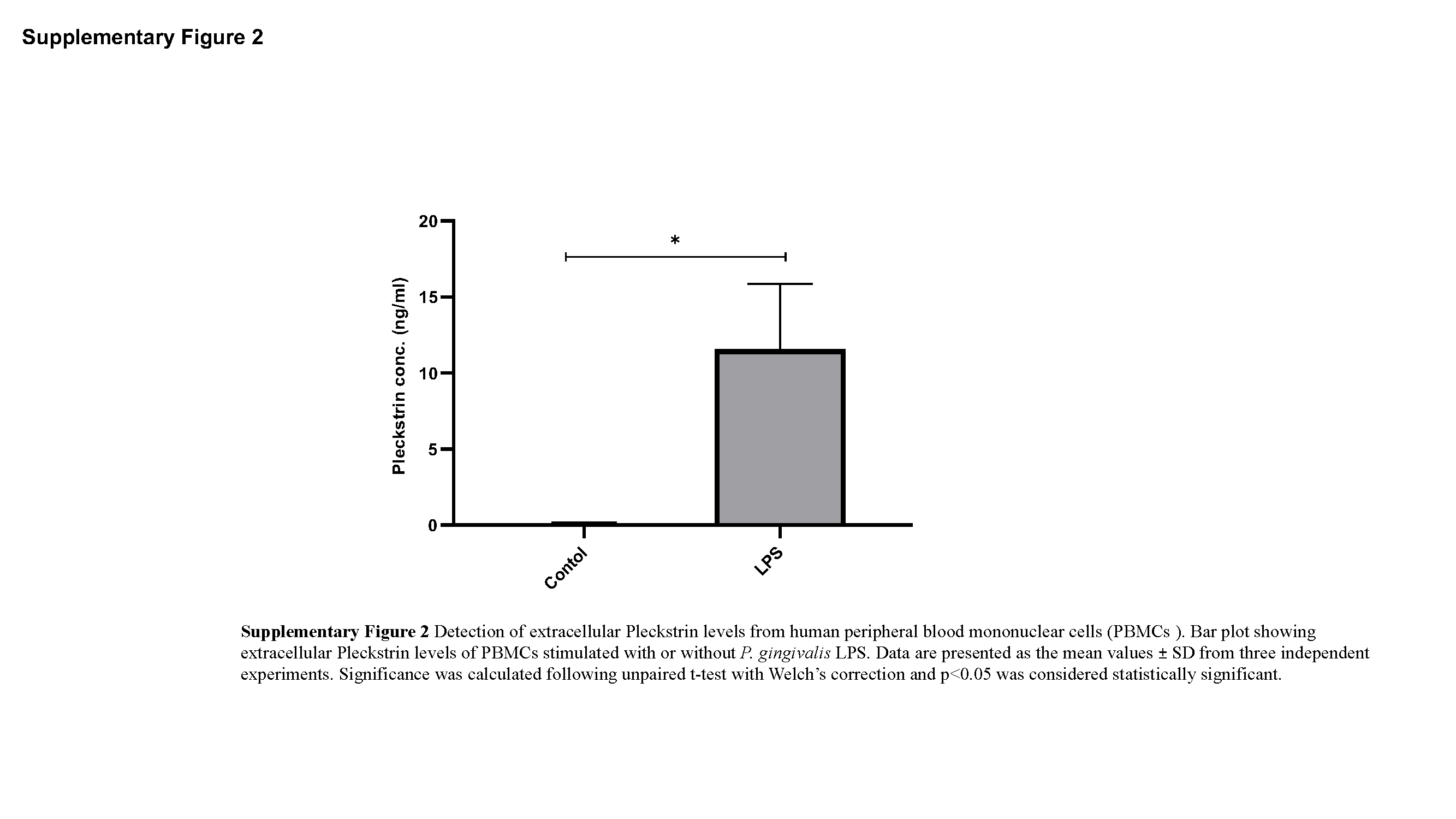

Supplement: Supplementary file 2 [file Image_2.tiff]

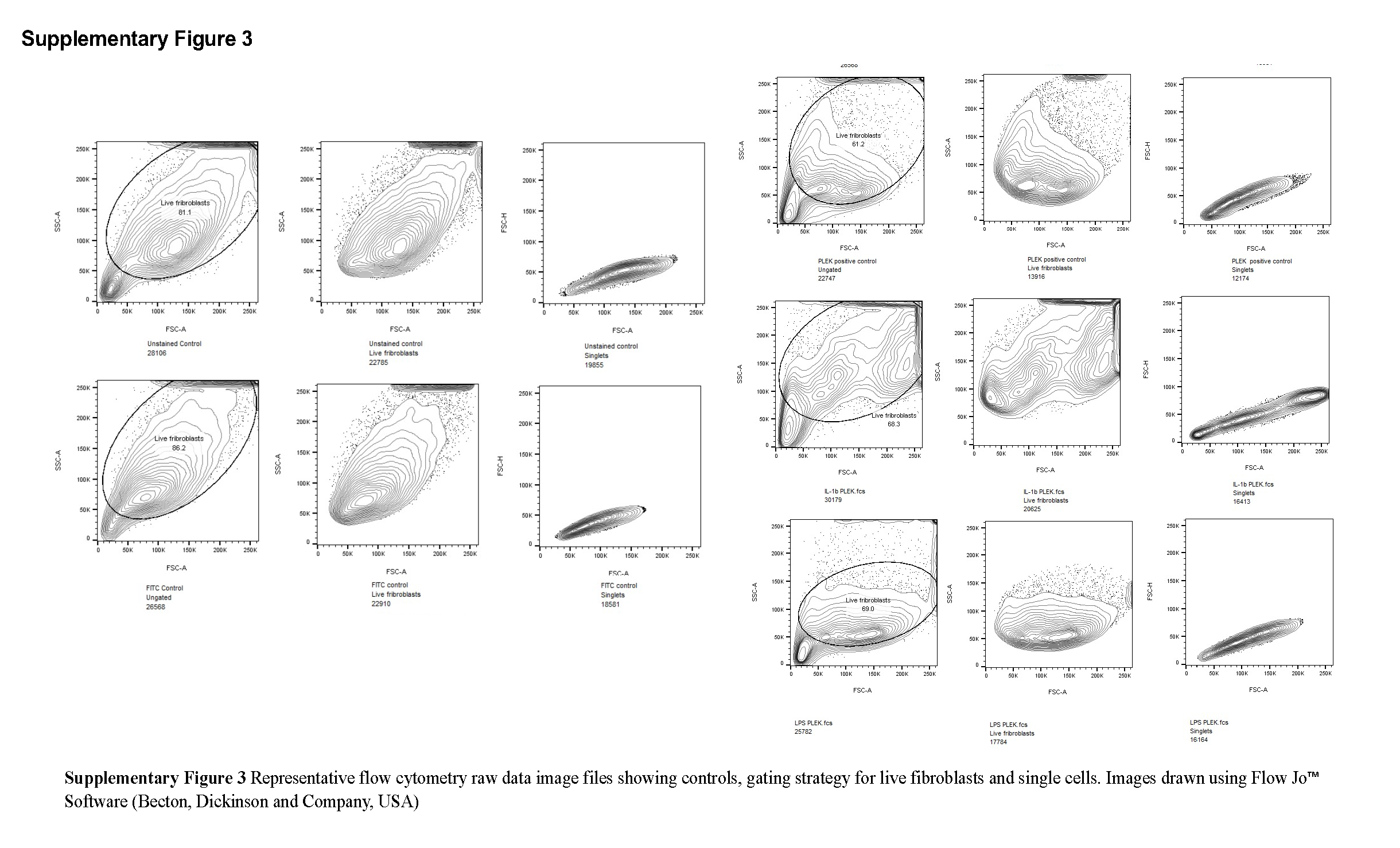

Supplement: Supplementary file 3 [file Image_3.tiff]
